# Acute‐Phase Interventions and Clinical Implementation Challenges for Hospital‐Associated Sarcopenia: A Narrative Review of a Multifaceted Approach to a Preventable Condition

**DOI:** 10.1111/ggi.70330

**Published:** 2026-01-21

**Authors:** Yoshinori Yamamoto, Masato Ogawa, Takayuki Okamoto, Marika Tsuboi, Ryo Momosaki

**Affiliations:** ^1^ Department of Rehabilitation Medicine Mie University Graduate School of Medicine Tsu Mie Japan; ^2^ Department of Rehabilitation Mie University Hospital Tsu Mie Japan; ^3^ Graduate School of Health Sciences Kobe University Kobe Hyogo Japan; ^4^ Department of Rehabilitation Kurashiki Rehabilitation Hospital Kurashiki Okayama Japan; ^5^ Department of Rehabilitation Medicine The Jikei University School of Medicine Shimbashi Tokyo Japan; ^6^ Department of Rehabilitation Medicine Tokyo General Hospital Nerima Tokyo Japan

**Keywords:** exercise therapy, hospitalization, muscle strength, nutrition therapy, sarcopenia

## Abstract

Hospital‐associated sarcopenia (HAS) is a preventable and reversible condition characterized by rapid muscle loss during hospitalization. Although its prevalence is higher than that of age‐related sarcopenia, the clinical recognition and structured management of this condition remain limited. In this narrative review, the pathophysiology of HAS is synthesized, the effectiveness of acute‐phase interventions is evaluated, and the implementation challenges are examined to propose multifaceted strategies for optimizing treatment outcomes. The development of HAS involves a vicious cycle of activity limitation, inflammation, malnutrition, and iatrogenic stress. Initiating interventions within 48 h may aid in preserving muscle function and improving the patients' quality of life. However, protocol variability, inadequate patient stratification, fragile transitional care systems, and other challenges persist. The proposed solutions include modular protocols, electronic medical record‐integrated adaptive algorithms, and strengthened team coordination. To prevent HAS progression and improve patient‐centered outcomes, timely, structured multidisciplinary interventions in the acute phase are imperative. Standardized evaluations, scalable protocols, and sustainable post‐discharge systems are key to advancing the clinical implementation of HAS management strategies.

## 1 Introduction

Sarcopenia is the age‐related decline in muscle mass and strength and is considered a major risk factor for falls and functional dependency in older adults [1]. This concept was first proposed by Rosenberg [1] and has since been further refined with diagnostic criteria established by the European Working Group on Sarcopenia in Older People [2] and the Asian Working Group for Sarcopenia [3].

Considerable research on the effects of acute illnesses and hospitalization on muscle function has elucidated the distinct concept of hospital‐associated sarcopenia (HAS) [4, 5]. HAS is defined as a rapid decline in muscle mass and strength during hospitalization caused by a combination of acute stressors, such as activity limitation, inflammation, malnutrition, and iatrogenic stress, often sustained during acute illness and medical interventions.

HAS is largely preventable and, in many cases, reversible. Its reversibility is evidenced by recovery from rapid skeletal muscle mass and strength deterioration with the resumption of physical activity and adequate protein–energy intake. In critically ill patients, substantial muscle atrophy begins in the first few days upon intensive care unit (ICU) admission. However, early mobilization and structured rehabilitation mitigate functional decline and shorten ICU stay [6, 7]. In healthy older adults, ~10 days of bed rest induces a ~10% reduction in lean mass and marked functional loss; however, these changes are generally reversed with the resumption of mobility and adequate nutritional intake [8, 9]. Collectively, these findings demonstrate that HAS is a transient and reversible form of secondary sarcopenia primarily driven by inactivity and inadequate nutrition, contrasting with the progressive and largely irreversible course of primary, age‐related sarcopenia [4, 5].

The prevalence of HAS is 14%–75% [10], which far exceeds that of age‐related sarcopenia at 10%–27% [4, 11]. Despite its high prevalence, HAS remains underrecognized in clinical settings. Furthermore, its systematic integration into clinical guidelines and assessment frameworks has not yet been established [12, 13]. These gaps are attributed to several factors, including difficulty in distinguishing HAS from age‐related sarcopenia, the lack of research examining acute‐phase muscle dysfunction, inconsistencies in diagnostic criteria, and fragmented evaluations of muscle mass, strength, and nutritional status [13, 14].

These challenges highlight the need for a multifaceted approach, including interventions initiated in acute‐phase hospitalization. Early identification and prevention of muscle deterioration are critical, considering patient vulnerability to rapid muscle function decline and systemic instability during this phase [15]. Timely exercise and nutritional interventions may aid in preserving muscle function, improving recovery trajectories, and reducing readmission rates [3, 16, 17].

Combining exercise and nutritional interventions may be more effective in mitigating HAS than either intervention alone. The successful implementation of this combined intervention in clinical settings requires interdisciplinary collaboration among rehabilitation professionals, dietitians, and physicians, as well as the development of transitional care plans to support patients following discharge [18, 19]. Moreover, outcome assessments should extend beyond considering just physical function but must also include quality of life (QOL), readmission rates, and other relevant indicators to facilitate individualized care.

Despite the growing awareness of HAS in clinical practice, few reviews have systematically addressed the difficulties in its clinical implementation or the phase‐specific strategies required for timely intervention within acute hospital care. Accordingly, the current review defines the distinctive clinical context of acute‐phase HAS and proposes practical, evidence‐based approaches to promote early identification, prevention, and integration of multidisciplinary evaluation into routine clinical practice.

## 2 Pathophysiology and Risk Factors of HAS

Pathophysiologically, HAS arises from the synergistic interaction of multiple hospital‐related stressors that accelerate skeletal muscle breakdown and impair recovery potential. These elements interact synergistically within the hospital environment to accelerate functional decline. Even brief periods of bed rest can already substantially reduce muscle function in otherwise healthy older and middle‐aged adults, highlighting the increased vulnerability to HAS during hospitalization [8, 20].

**FIGURE 1:**
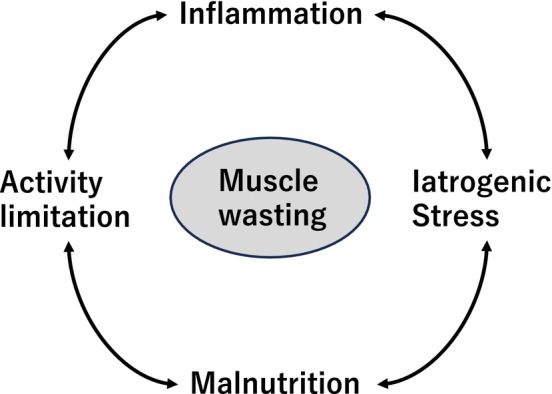
Conceptual model of the vicious cycle underlying hospital‐associated sarcopenia. This schematic illustrates the key risk factors contributing to muscle wasting during hospitalization. The interplay of activity limitation, inflammation, malnutrition, and iatrogenic stress creates a self‐perpetuating cycle that accelerates sarcopenia progression.

Hospital‐specific stressors and underlying mechanisms interact to form a vicious cycle in which each factor amplifies the others, leading to progressive muscle atrophy and functional decline during hospitalization (Figure 1).

### 2.1 Activity Limitation and Muscle Function Decline

Physical inactivity is a major, modifiable contributor to muscle loss in hospitalized patients. Prolonged bed rest inhibits the mechanistic target of rapamycin signaling pathway and activates the ubiquitin–proteasome system, resulting in decreased muscle protein synthesis and accelerated muscle degradation [21]. These catabolic effects are exacerbated by systemic inflammation and age‐related anabolic resistance.

In older adults, up to 10% of muscle mass and 13% of muscle strength can be lost within just 10 days of inactivity [8]. Patients with critical illness may incur as high as 20% muscle loss within the first 48 h of admission to the ICU [6]. Furthermore, extremely low physical activity levels, such as walking < 900 steps per day, and a prolonged sedentary condition significantly reduce mobility and increase the risk of hospital readmission [18, 22].

### 2.2 Inflammation and Muscle Metabolic Dysregulation

Acute systemic inflammation disrupts skeletal muscle metabolism and promotes catabolic processes. Proinflammatory cytokines, such as interleukin‐6 (IL‐6) and tumor necrosis factor alpha, activate the Forkhead box O3a signaling pathway, which induces the upregulation of muscle‐specific E3 ubiquitin ligases, including muscle really interesting new gene‐finger protein 1 and atrogin 1 [23]. Concurrently, the nuclear factor kappa‐light‐chain‐enhancer of the activated B‐cell pathway promotes anabolic resistance, impairing muscle protein synthesis.

Persistently elevated IL‐6 levels are strongly predictive of muscle atrophy and have been associated with pronounced atrophy in patients with severe COVID‐19 [24].

### 2.3 Nutritional Impairment and Metabolic Stress

Nutritional deficiencies during hospitalization further accelerate muscle degradation. Contributing factors include prolonged fasting, reduced oral intake, medication side effects, and dysphagia, particularly in patients with low Mini Nutritional Assessment (MNA) scores, who are at an increased risk of rapid muscle decline [25].

Anabolic resistance, which is commonly observed in aging and acute illness, further impairs muscle protein synthesis. Catabolic states, such as sepsis or cancer, which increase basal energy expenditure, accompanied by insufficient nutrient intake, further aggravate muscle atrophy and functional decline [26].

### 2.4 Medical Invasiveness and Length of Stay

Invasive medical interventions, such as mechanical ventilation, catheterization, and major surgery, inevitably limit physical activity and increase metabolic stress, thereby accelerating the development of sarcopenia in hospitalized patients [27, 28, 29]. Prolonged hospitalization (> 10 days) and extended ICU stay (> 5 days) are strongly associated with substantial muscle loss and adverse clinical outcomes [6, 30].

Moreover, environmental stressors commonly encountered in hospital settings, such as excessive noise, sleep disruption, and cognitive overload, contribute further to muscle degradation and delayed recovery [31].

### 2.5 Vulnerability to Aging and Underlying Conditions

Aging and chronic illnesses increase metabolic vulnerability by progressing type II muscle fiber atrophy, hormonal dysregulation, and diminished regenerative capacity. Patients with underlying conditions, such as liver disease or malignancies, are especially susceptible to accelerated muscle loss and poor functional outcomes [32, 33, 34]. These patients should be considered pre‐sarcopenic upon hospital admission and prioritized for early screening and proactive preventive strategies.

### 2.6 Clinical and Epidemiological Contexts of HAS

HAS manifests across a wide range of acute and chronic medical conditions, which highlights the converging influences of systemic inflammation, immobility, and malnutrition. Incidence rates vary markedly from 14% to 75%, depending on patient characteristics, diagnostic definitions, and clinical settings [10].

Among hospitalized populations, older in‐patients are particularly vulnerable, especially those with acute illnesses, such as pneumonia, systemic infection, heart failure, or malignancy, where inflammation‐driven catabolism and prolonged bed rest accelerate skeletal muscle degradation. The incidence is ~14%–25% among geriatric inpatients, with low body mass index (BMI) and immobility emerging as key independent predictors [11]. Patients with liver cirrhosis or those undergoing organ transplantation are also comparably vulnerable, owing to hypermetabolism, hypoalbuminemia, and postoperative complications, such as infection or renal dysfunction, each of which accelerates muscle loss [27, 35].

In surgical cohorts, HAS frequently develops in patients undergoing colorectal, abdominal, or hip fracture surgery, which are characterized by postoperative immobility and inadequate nutritional intake, impeding recovery [5, 13, 36]. Sarcopenia develops in roughly 20% of patients undergoing cardiac surgery, which is associated with prolonged mechanical ventilation, diabetes, and low BMI [28]. Likewise, individuals with esophagogastric cancer experience pronounced reductions in lean body mass following neoadjuvant chemotherapy, even preoperatively [37]. Among COVID‐19 survivors, persistent systemic inflammation, and extended ICU stays have been linked to enduring sarcopenia and malnutrition [38].

Across these diverse clinical contexts, advanced age, low BMI, frailty, and longer hospital stays consistently emerge as independent risk factors. Thus, HAS should be clinically regarded as a secondary but preventable complication that arises in response to disease‐specific stressors. Therefore, early screening and personalized, multidisciplinary care are essential for at‐risk patients.

Table 1 summarizes key studies on the clinical and epidemiological features of HAS across medical and surgical populations.

**TABLE 1:** Representative studies reporting the clinical and epidemiological characteristics of HAS across medical and surgical populations.

Author, year	Study population	Diagnostic of sarcopenia	Timing of assessment	Prevalence of sarcopenia	Risk factors
Martone et al. (Italy, 2017)	394 patients (mean age, 79.6 ± 6.4 years) admitted to geriatric and internal medicine acute care wards	EWGSOP	At admission/At discharge (mean days 10.2 ± 8.1)	58/394 (14.7%)	Number of days spent in bed, Low body mass index
Yuenyongchaiwat et al. (Thailand, 2020)	153 preoperative open‐heart surgery patients (mean age, 61.1 ± 11.5 years)	AWGS	Before surgery/Before discharge	32/153 (20.9%)	Age, Low body mass index, Diabetes mellitus, Mechanical ventilation times, Length of stay
Malafarina et al. (Spain, 2019)	187 patients (mean age, 85.2 ± 6.3 years) were admitted for rehabilitation after hip fracture surgery	EWGSOP	At admission/At discharge	54/187 (28.8%)	Low body mass index, Mini Nutritional Assessment‐short form, High TNFs, Barthel index before hospitalization
Welch et al. (UK, 2019)	7 patients (mean age, 74.7 ± 4.1 years) undergoing elective colorectal surgery	EWGSOP	Pre‐surgery baseline/1 week after colorectal cancer surgery or at discharge	6/8 (75%)	hsCRP
Welch et al. (UK, 2022)	81 patients (mean age, 79.2 ± 6.6 years) admitted for elective colorectal surgery, emergency abdominal surgery, or acute infections	Skeletal muscle mass (Bilateral Anterior Thigh Thickness and/or bioelectrical impedance analysis)	At baseline/7 (±2) days post‐hospitalisation or post‐surgery, 13 (±1) weeks post‐hospitalisation or post‐surgery	5/25 (20.0%)	Gait speed
Levy et al. (France, 2022)	139 patients with COVID‐19 (median age, 62 (29–82) years) admitted to the ICU and/or the pulmonology department	EWGSOP2	At admission/3 and 6 months after discharge	22/139 (15.8%)	Length of stay, Intensive care unit length of stay, Tracheostomy
Nguyen et al. (Canada, 2006)	79 patients with cirrhosis (mean age, 54 ± 11 years) undergoing liver transplantation	Skeletal muscle mass (CT)	Before liver transplantation/3 months after liver transplantation	25/161 (15.5%)	Length of stay, Number of infection, Number of complications
Bhanji et al. (USA, 2019)	293 liver transplant recipients (mean age, 51.9 ± 11 years)	Skeletal muscle mass (CT)	Before liver transplantation (median 9.6 months)/after surgery (median 7.2 months)	18/43 (41.8%)	Complications
Awad et al. (UK, 2012)	47 patients with esophagogastric cancer (mean age, 63 ± 12 years)	Skeletal muscle mass (CT)	Before chemotherapy/after chemotherapy	10/47 (21.2%)	Low fat‐free mass

*Note:* This table summarizes representative studies investigating the prevalence, diagnostic criteria, timing of assessment, and major risk factors of HAS in medical and surgical populations. The data illustrate substantial variability in reported prevalence (14%–75%), largely reflecting differences in patient characteristics, diagnostic definitions, and clinical settings. Common determinants include low body mass index, immobility, inflammation, malnutrition, and prolonged hospitalization, underscoring the multifactorial nature of HAS and the need for early, integrated interventions.

## 3 Strategic Acute‐Phase Interventions and Challenges

Acute‐phase interventions are critical for preventing HAS progression. In the early stage of hospitalization, patients show increased vulnerability to muscle loss but also increased responsiveness to therapeutic interventions. This paradox is supported by clinical and physiological evidence indicating that early mobilization and nutritional support sustain muscle protein synthesis while attenuating catabolic signaling during hospitalization.

Early rehabilitation improves functional outcomes and reduces complications associated with muscle loss and immobilization [7]. Resistance training (RT) and neuromuscular electrical stimulation (NMES) can also help preserve muscle mass, even in patients with limited mobility [39, 40, 41, 42]. Likewise, adequate protein intake and targeted supplementation help counteract catabolic processes [43, 44, 45, 46]. Overall, the integration of exercise and nutritional strategies provides synergistic benefits for muscle metabolism and recovery, as demonstrated by mechanistic and clinical evidence [22, 47].

The timely implementation of exercise and nutritional strategies not only helps preserve muscle function but also improves key clinical outcomes, including activities of daily living (ADL) and QOL.

The next section discusses the clinical utility, limitations, and challenges in implementing exercise and nutritional interventions, both as stand‐alone and combined approaches.

### 3.1 Physical Activity and Exercise Interventions

Physical inactivity is a central, modifiable risk factor for HAS. During ICU admission and postoperatively, even short‐term bed rest impairs muscle function and functional status. Thus, early mobilization within 48 h of ICU admission is critical. For patients with critical illness, 20‐min sessions of physical and occupational therapy, once or twice daily, can reduce mechanical ventilation time and hospital stay while improving ADL [7].

Tailored interventions based on functional capacity are essential. This is implemented in practice by stratifying patients according to their functional status at admission. For bedridden patients with little or no voluntary movement, NMES (30–50 Hz) can be employed to prevent muscle atrophy and promote circulation [41, 42]. Patients capable of sitting or briefly standing can undergo early mobilization therapies, such as supervised bedside activities and short sessions of assisted ambulation [7]. Those with higher functional capacity can engage in RT at 60%–70% of one‐repetition maximum two to three times per week to improve muscle mass, grip strength, and gait speed [39, 40]. This stepwise approach provides a practical framework for safely and effectively individualizing exercise interventions during acute‐phase hospitalization. However, the implementation of RT and NMES in hospitalized patients is often limited by variations in patient condition severity and institutional resources, highlighting the practical challenges of applying these mobilization strategies in routine clinical settings.

### 3.2 Nutritional Interventions

Malnutrition is a major driver of HAS, particularly during early hospitalization, when reduced appetite, dysphagia, drug effects, and fasting accelerate nutritional decline. Current guidelines for muscle preservation recommend 1.2–1.5 g/kg/day of protein, supported by targeted supplementation with branched‐chain amino acids (BCAA), β‐hydroxy‐β‐methylbutyrate (HMB), vitamin D, and omega‐3 fatty acids [43, 44, 45, 46]. Continued supplementation with HMB (3 g/day, orally or enterally) for at least 12 weeks promotes improvement in muscle mass and gait speed [46, 47]. Oral nutritional supplements that provide ~400–600 kcal and 20–30 g of protein per day for at least 12 weeks have been shown to enhance muscle strength and physical performance in malnourished or nutritionally at‐risk hospitalized patients [48]. These nutrients are utilized through anabolic, anti‐inflammatory, or functional mechanisms to improve muscle mass, strength, and functional outcomes, especially in older or immobilized patients [47, 49, 50].

To translate these findings into practice, nutritional management during acute care should be implemented through a stepwise escalation pathway based on current international guidelines [43, 44]. Oral intake with enriched meals and oral nutritional supplements serves as the first‐line approach. If < 60% of nutritional requirements are acquired within 48 h, enteral nutrition should be initiated, provided gastrointestinal function is preserved. If enteral feeding is contraindicated or remains insufficient after 72 h, parenteral nutrition should be introduced to ensure adequate energy and protein delivery [51]. Screening with standardized tools, such as the MNA–short form or nutritional risk screening–2002, should be performed within 24 h of admission, with oral or enteral support initiated within 48 h and escalation considered by 72 h if protein goals remain unmet [51, 52].

Establishing a multidisciplinary nutritional support team (NST) is pivotal in ensuring structured, coordinated, and timely nutritional care to prevent HAS. A standard NST typically includes a physician, dietitian, nurse, and pharmacist, and often collaborates with rehabilitation specialists and speech‐language pathologists for patients with dysphagia or impaired oral intake.

Each professional provides complementary expertise: Physicians assess nutritional safety and determine the appropriate route of administration, while dietitians establish individualized protein and energy targets (typically 1.2–1.5 g protein/kg body weight/day) and design supplementation plans. Nurses monitor daily intake, tolerance, and feeding complications, while pharmacists evaluate potential drug–nutrition interactions. Finally, rehabilitation specialists synchronize exercise timing with nutrient delivery to maximize anabolic efficiency and enhance functional recovery [52, 53].

Early activation of NSTs ensures the prompt initiation of oral or enteral nutrition, minimizes unnecessary parenteral feeding, and guarantees nutritional adequacy and care continuity. NST engagement within 24 h of admission and standardized enteral feeding within 72 h can improve protein delivery, reduce complications, and shorten hospital stays among acutely ill older adults [53, 54].

Beyond direct nutrition management, NSTs initiate and sustain organizational impetus in coordinating early screening, risk stratification, escalation of nutritional support, and rehabilitation to prevent HAS and expedite recovery [55, 56]. Hospitals implementing structured NST programs demonstrate higher adherence to clinical nutrition guidelines, better documentation, and more consistent multidisciplinary coordination across care transitions [57].

Collectively, these findings highlight the essential contribution of NSTs in integrating nutrition therapy and rehabilitation to promote early anabolic stimulation and integrated care during acute care, when the prevention of muscle wasting is most critical.

### 3.3 Combined Interventions and Clinical Integration

Given the multifactorial nature of HAS, the combination of exercise and nutritional strategies may exert synergistic effects toward its prevention or minimization.

The concept of anabolic timing, which involves providing nutrients during the post‐exercise period of heightened metabolic sensitivity, represents a practical strategy for enhancing recovery [2, 23]. Intake of 20–40 g of rapidly digestible, leucine‐rich protein within 30–60 min after exercise maximizes muscle protein synthesis, particularly in older adults with anabolic resistance [43, 58]. Practical options include oral supplementation with whey, BCAA, or HMB immediately after rehabilitation sessions, as such nutrients enhance post‐exercise anabolism and recovery [43, 44, 45, 46, 59]. Combining enteral feeding protocols with exercise may also optimize nutrient utilization in less mobile or critically ill patients. Transitional care models for supporting nutrition, medication, and physical activity coordination may lower readmission rates [60], although the heterogeneity in protocols and patient selection hinders reproducibility and generalization [23, 61].

Successful implementation of individualized, phase‐specific interventions requires constant interdisciplinary collaboration among rehabilitation professionals, dietitians, nurses, and physicians. This process can be supported by structured quality improvement frameworks, such as the PDCA (plan–do–check–act) cycle [16]. To enhance the clinical applicability of the PDCA framework, explicit outcome indicators and stratification criteria should be implemented to guide both monitoring and individualized intervention [62].

Functional outcomes can be objectively assessed using the Barthel Index, which measures performance in ADL such as feeding, bathing, and mobility. Scores below 60 indicate marked dependency, whereas an improvement of ≥ 10 points during hospitalization denotes meaningful functional recovery [63]. Nutritional status should be evaluated using the MNA‐short form, for which scores < 8 denote high nutritional risk [64]. Patients with prolonged ICU stays (> 5 days), severe comorbidities (Charlson Comorbidity Index ≥ 5), or low physical performance (gait speed < 0.8 m/s or handgrip strength < 20 kg for women and < 30 kg for men) are considered at high‐risk and should thus receive early, combined rehabilitation and nutrition interventions [65, 66, 67].

These objective thresholds facilitate patient stratification, enable timely adaptation of care plans, and provide measurable outcomes for progress monitoring throughout hospitalization. Integrating these structured assessments within the PDCA cycle promotes continuous feedback, individualized adjustment, and multidisciplinary coordination, which ultimately enhances functional recovery and reduces readmission risk [62, 68]. The interventions should be stratified based on patient condition and tailored to the hospitalization phase (Table 2). For example, NMES combined with blood flow restriction may be appropriate for patients with critical illness, whereas combining RT with BCAA supplementation may benefit those with moderate physical function.

**TABLE 2:** Fundamental strategies for the design of intervention for hospital‐associated sarcopenia.

Intervention goal	Recommended intervention	Recommended initiation	Frequency/duration	Remarks
Maintenance of muscle mass	Neuromuscular electrical stimulation, resistance training, blood flow restriction	Within 48 h of admission	1–2 times/day; 20–45 min/session, 2–3 sessions/week	Applicable even in patients without voluntary movement
Improvement of muscle function	Resistance training, moderate‐intensity aerobic exercise	Promptly after stabilization	2–3 times/week, ≥ 30 min	Approximately 60%–70% of one‐repetition maximum
Improvement of nutritional status	Protein, β‐hydroxy β‐methylbutyrate, branched‐chain amino acid supplementation	Immediately after admission	Daily, target 1.2–1.5 g/kg/day	Adapted to the swallowing function and patient preferences
Enhancement of activities of daily living and quality of life	Combined interventions (exercise + nutrition) plus psychological support	Mid‐hospitalization to post‐discharge	Continuous	Coordination with the family and community is essential

*Note:* This table summarizes the representative interventions for hospital‐associated sarcopenia, classified according to their clinical objectives, and organized with corresponding initiation timing, frequency, and key considerations for implementation. It provides fundamental guidance for the practical design of intervention programs.

**FIGURE 2:**
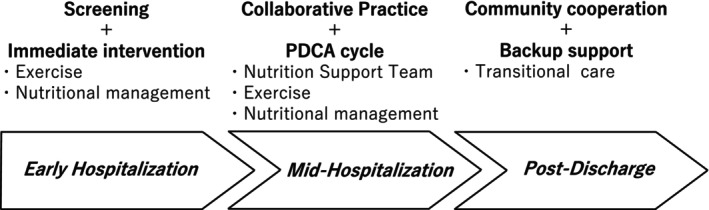
A phased clinical intervention model for hospital‐associated sarcopenia. This framework outlines a three‐stage intervention strategy: early hospitalization (screening and immediate initiation of exercise and nutritional support), mid‐hospitalization (multidisciplinary care and PDCA‐cycle implementation), and post‐discharge (community‐based collaboration and transitional care). The model emphasizes continuity and individualization to prevent functional decline and optimize recovery.

After discharge, coordinated community‐based support systems are crucial for maintaining functional gains. Stratified intervention approaches can help optimize treatment responsiveness and ensure individualized care (Figure 2).

## 4 Clinical Barriers and Strategic Solutions

Despite the reported benefits of acute‐phase interventions for HAS, multiple barriers hinder their clinical implementation. Operational and systemic strategies must be implemented to overcome these barriers. This section outlines the major challenges and potential solutions.

### 4.1 Early Intervention and Patient Selection

Muscle loss occurs rapidly during the first few days of hospitalization, highlighting the importance of the timely implementation of mobilization, RT, NMES, and nutritional support. However, standardized criteria to guide “who receives what, when” are lacking. Furthermore, assessment tools, such as the Barthel Index and MNA, are used inconsistently and often without integration into care pathways [69, 70, 71].

To address these limitations, real‐time evaluation systems that incorporate multifactorial scoring algorithms for assessing physical function, nutritional status, and comorbidities should be developed [15].

### 4.2 Protocol Variability and Coordination Gaps

Exercise and nutritional interventions vary across facilities in terms of content, frequency, and staffing, creating challenges in balancing protocol standardization with patient‐specific adaptability [40, 72]. Thus, structured workflows are required to operationalize conceptual strategies, such as modular protocols, electronic medical record (EMR)‐linked alerts, and PDCA cycles. EMR‐based screening upon admission can automatically identify high‐risk patients (e.g., Barthel Index < 60, MNA < 8) and initiate bundled orders for early mobilization, NMES, and NST consultation. Early, protocolized mobilization and nutritional interventions reduce ICU‐acquired weakness and improve functional outcomes [73, 74]. Although early mobilization, NMES, and high‐protein intake are well supported by clinical evidence, EMR‐based modular order sets remain conceptual and require empirical validation. Defining the roles of coordinators and standardizing documentation can strengthen integration. In practice, establishing mobility teams and nurse‐led collaboration helps minimize delays in exercise initiation, whereas early NST engagement and tailored nutrition improve overall care. PDCA cycles and EMR‐based communication platforms can decisively synchronize exercise and nutritional strategies. Regardless of institutional variations, these pragmatic approaches can enhance reproducibility, ensure quality, and improve outcomes, even in resource‐limited environments (Table 3).

**TABLE 3:** Key components, implementation challenges, and suggested strategies for multifaceted interventions for hospital‐associated sarcopenia.

Intervention domain	Key components	Challenges	Strategic solutions
Physical activity and exercise	Early mobilization, resistance training, and neuromuscular electrical stimulation	Variability across facilities; limited staffing; difficulty ensuring continuity	Standardized rehabilitation protocols; dedicated mobility teams; early rehabilitation education
Nutritional management	Protein supplementation, β‐hydroxy β‐methylbutyrate supplementation, and collaboration with nutritional support teams	Difficulties with intake; suboptimal timing and dosage	Active involvement of nutritional support teams; enriched oral nutritional supplements; swallowing assessment, and texture modification
Combined interventions	Anabolic timing strategies and integrated intervention models	Heterogeneity in intervention composition; poor reproducibility	Modular intervention packages; synchronized meal and rehabilitation schedules
Post‐discharge support	Transitional care; coordination with community‐based care systems	Inadequate information‐sharing; inconsistency in outcome evaluation	Care coordinators; standardized discharge summaries; and strengthened community liaison systems
Implementation evaluation	Monitoring of fidelity, dosage, and adaptation; tracking of quality of life and readmission rates	Need for multilayered evaluation systems; integration with reimbursement frameworks	Bundled outcome indicators; electronic medical record‐linked dashboards; and policy advocacy

*Note:* This table presents the key domains of multifaceted interventions for hospital‐associated sarcopenia, highlighting the core components, representative implementation challenges, and practical examples of strategic solutions. By linking challenge identification with actionable strategies, the framework provides practical guidance to enhance the reproducibility and clinical effectiveness of the intervention models.

### 4.3 Post‐Discharge Support and Outcome Evaluation

Post‐discharge care is pivotal for sustaining muscle recovery and preventing worsening of HAS. Rather than generalized discharge planning, HAS‐specific strategies should be emphasized, including early post‐acute rehabilitation, structured home‐based exercise regimens, and nutritional follow‐up, which are optimally coordinated through community care systems.

Recent evidence demonstrates the effectiveness of community‐integrated interventions that combine physical rehabilitation, nutritional counseling, and social support to facilitate functional recovery and reintegration after hospitalization. Such multidisciplinary approaches have prompted improved physical performance, reduced rehospitalization, and shorter subsequent hospital stays among older adults in community settings [18, 53, 60].

Collectively, these findings demonstrate the importance of coordinated post‐discharge programs that extend the benefits of acute‐phase interventions and support long‐term recovery in individuals vulnerable to HAS. Aligning post‐discharge care with HAS prevention frameworks may help maintain the anabolic trajectory initiated during hospitalization, such as through early outpatient reassessment, continued RT and protein‐based interventions, as well as close collaboration maintained among hospitals, primary care, and community services.

### 4.4 Limitations

This review did not follow a fully systematic or scoping search methodology. Although we attempted to capture key and representative evidence on acute‐phase interventions for HAS, some relevant studies may have been missed (e.g., due to variability in terminology, database coverage, or unpublished work). The narrative design was deliberately applied because the existing evidence is heterogeneous, emerging, and not yet amenable to quantitative synthesis. Therefore, our aim was ultimately to provide a conceptual, clinically driven, and multidisciplinary integration of the subject, given the unavailability of comprehensive evidence mapping or meta‐analysis.

## 5 Conclusion

HAS is not an inevitable result of aging but rather a preventable and reversible condition driven by transient environmental stressors during hospitalization. This review investigated the pathophysiology of HAS, the effectiveness of acute‐phase exercise and nutritional interventions, and key implementation challenges.

The development of HAS involves a self‐perpetuating vicious cycle—activity limitation, inflammation, malnutrition, and iatrogenic stress—that occurs in early hospitalization. The acute phase provides a strategic window for intervention, during which multidisciplinary approaches can improve ADL and QOL as well as reduce readmissions.

Successful implementation of HAS management strategies requires standardized outcome measures, adaptable protocols, clear team roles, and sustainable post‐discharge support. These efforts are clinically and socially vital to the preservation of dignity and independence.

We propose a multidisciplinary model spanning from early hospitalization to discharge. The key steps of this model include early screening within 48 h, timely bedside interventions, and integrated planning among healthcare professionals. These steps should evolve into structured assessments, discharge conferences, and coordinated community‐based care.

Maximization of this narrow intervention window and alignment of clinical strategies with structural systems will be crucial for effective HAS prevention.

## Author Contributions

**Yoshinori Yamamoto:** conceptualization, data curation, investigation, methodology, project administration, resources, validation, visualization, writing – original draft, writing – review and editing. **Masato Ogawa:** conceptualization, investigation, methodology, validation, visualization, writing – review, and editing. **Takayuki Okamoto:** conceptualization, investigation, methodology, validation, writing – review and editing. **Marika Tsuboi:** conceptualization, investigation, methodology, validation, writing – review and editing. **Ryo Momosaki:** conceptualization, supervision, validation, writing – review and editing.

## Funding

The authors have nothing to report.

## Conflicts of Interest

The authors declare no conflicts of interest.

## Data Availability

The data that support the findings of this study are available from the corresponding author upon reasonable request.
